# Actin-Related Protein Arp6 Influences H2A.Z-Dependent and -Independent Gene Expression and Links Ribosomal Protein Genes to Nuclear Pores

**DOI:** 10.1371/journal.pgen.1000910

**Published:** 2010-04-15

**Authors:** Takahito Yoshida, Kenji Shimada, Yukako Oma, Véronique Kalck, Kazumi Akimura, Angela Taddei, Hitoshi Iwahashi, Kazuto Kugou, Kunihiro Ohta, Susan M. Gasser, Masahiko Harata

**Affiliations:** 1 Laboratory of Molecular Biology, Graduate School of Agricultural Science, Tohoku University, Sendai, Japan; 2 Friedrich Miescher Institute for Biomedical Research, Basel, Switzerland; 3 Centre National de la Recherche Scientifique/Institut Curie-Section de Recherche, Paris, France; 4 Human Stress Signal Research Center, National Institute of Advanced Industrial Science and Technology, Tsukuba, Ibaraki, Japan; 5 Shibata Distinguished Senior Laboratory, RIKEN Discovery Research Institute, Wako, Saitama, Japan; 6 Department of Life Sciences, Graduate School of Arts and Sciences, The University of Tokyo, Tokyo, Japan; Medical Research Council Human Genetics Unit, United Kingdom

## Abstract

Actin-related proteins are ubiquitous components of chromatin remodelers and are conserved from yeast to man. We have examined the role of the budding yeast actin-related protein Arp6 in gene expression, both as a component of the SWR1 complex (SWR-C) and in its absence. We mapped Arp6 binding sites along four yeast chromosomes using chromatin immunoprecipitation from wild-type and *swr1* deleted (*swr1Δ*) cells. We find that a majority of Arp6 binding sites coincide with binding sites of Swr1, the catalytic subunit of SWR-C, and with the histone H2A variant Htz1 (H2A.Z) deposited by SWR-C. However, Arp6 binding detected at centromeres, the promoters of ribosomal protein (RP) genes, and some telomeres is independent of Swr1 and Htz1 deposition. Given that RP genes and telomeres both show association with the nuclear periphery, we monitored the ability of Arp6 to mediate the localization of chromatin to nuclear pores. Arp6 binding is sufficient to shift a randomly positioned locus to nuclear periphery, even in a *swr1Δ* strain. Arp6 is also necessary for the pore association of its targeted RP promoters possibly through cell cycle-dependent factors. Loss of Arp6, but not Htz1, leads to an up-regulation of these RP genes. In contrast, the pore-association of *GAL1* correlates with Htz1 deposition, and loss of Arp6 reduces both *GAL1* activation and peripheral localization. We conclude that Arp6 functions both together with the nucleosome remodeler Swr1 and also without it, to mediate Htz1-dependent and Htz1-independent binding of chromatin domains to nuclear pores. This association is shown to have modulating effects on gene expression.

## Author Summary

Actin and the structurally similar actin-related proteins (ARPs) are major components of nucleosome remodeling complexes in the nucleus. Here we show that budding yeast Arp6 has functions independent of its catalytic chromatin remodeling partner, Swr1. Arp6 binds to multiple promoters and subtelomeric zones at which Swr1 does not bind, and this association increases in strains lacking an intact SWR-C remodeling complex. We show that Arp6 can mediate the association of the ribosomal protein gene promoters to which it binds, with the nuclear envelope. The loss of Arp6 led to an up-regulation of these ribosomal protein genes, yet had the opposite effect on the galactose-induced *GAL1* promoter, where it binds together with Swr1 and the histone H2A variant H2A.Z. Indeed, loss of Arp6 and nuclear envelope binding delays galactose-induced activation of *GAL1*. The two opposing functions of Arp6 correlate with different pathways of anchoring mediated by Arp6: one requires that the intact SWR-C deposits H2A.Z, while in the second Arp6 may mediate promoter binding to nuclear pore baskets directly. In both cases, Arp6 is implicated in the spatial localization of chromatin within the interphase nucleus, which has functional consequences on expression. This identifies a novel function for actin-related proteins.

## Introduction

Genomic DNA is complexed with histones and non-histone proteins to form chromatin, which is organized into active and inactive domains within the interphase nucleus [1]–[3]. Histone tail modifications, chromatin compaction, and the subnuclear positioning of chromatin domains contribute epigenetic information that helps to determine gene expression patterns. While the enzymology of histone modification has been well characterized, little is known about the mechanisms that determine the spatial organization of chromatin in interphase nuclei.

In both vertebrates and yeast, transcriptionally inactive heterochromatin is enriched around the nucleolus or at the nuclear envelope (NE). In vertebrates, perinuclear anchoring appears to require the nuclear lamina, while in yeast integral proteins of the inner nuclear membrane tether repressed chromatin domains peripherally [reviewed in 4]. Recent work has shown that in addition to silent heterochromatic loci, some euchromatic yeast genes are found at the NE as well. Indeed, inducible budding yeast genes such as *INO1*, *GAL1*, and *HXK1* form a stable association with the nuclear pore complex (NPC) upon activation. In some cases, this interaction ensures maximal expression and fine-tuning of induction rates [5]–[7]. The up-regulated X chromosome in male flies may also be associated with nuclear pores [8], as are the highly transcribed ribosomal protein (RP) genes of yeast [9].

Besides nuclear pore proteins, little is known about the components that position active chromatin domains within the nucleus. Nuclear actin and myosin, as well as myosin-like and actin-related proteins have been proposed as candidates that could contribute to the organization of transcription in the interphase nucleus [8], [10]–[17]. Indeed, actin itself is not only found as part of the filamentous cytoskeleton, but in various large chromatin modifying complexes, which are exclusively nuclear.

In all organisms from yeast to man, the actin family includes a number of proteins that are structurally similar to actin, called actin-related proteins or ARPs. The yeast *S. cerevisiae* alone harbors ten *ARP* genes, numbered from 1 to 10, with ascending degrees of dissimilarity to actin [18]–[19]. Arp1-3 and Arp10 are cytoplasmic and help regulate cytoskeletal structures, while the other six (Arp4 to Arp9) are nuclear proteins [20]–[23]. Nuclear ARPs, like nuclear actin, are often found in ATP-dependent chromatin modifying complexes that shift or displace nucleosomes, or in complexes that acetylate histone tails (e.g. NuA4 complex) [reviewed in 23]. Exactly how ARPs contribute to nucleosome modification, however, is unknown.

Arp6 is an evolutionarily conserved nuclear ARP [24]–[26]. The budding yeast Arp6, along with two other actin-family members, Act1 and Arp4, are part of the 14-component SWR1 chromatin remodeling complex (SWR-C), which is called SRCAP or Snf2-Related CREB-binding Activator Protein in mammals [27]. In addition to the ATPase subunit, Swr1, SWR-C includes Swc1/Fun36, Swc2/Vps72, Swc3, Swc4/God1, Swc5/Aor1, Swc6/Vps71, Swc7, Yaf9, Bdf1, Rvb1, and Rvb2 [28]. The SWR-C holocomplex can exchange H2A with its variant H2A.Z (Htz1 in budding yeast) in assembled nucleosomes [29], [30]. Arp6 appears to form a subcomplex with Swc2, Swc3, and Swc6, and helps bridge this subcomplex with a second one containing Swr1, to form the functional SWR-C [28]. Since Swc2 component is responsible for binding Htz1, the Arp6-mediated bridging is necessary for Htz1 deposition [28], [29].

Nucleosomes containing acetylated H2A.Z are specifically enriched at promoters in higher eukaryotes, and consistently, Htz1 is found in nucleosomes flanking the nucleosome-free region located at transcription start sites in yeast [31]–[36]. Additionally, Htz1 prevents the spreading of heterochromatin proteins in subtelomeric domains [35], [37], [38]. *In vitro* analyses have indicated that Arp6 contributes to transcriptional regulation by mediating Htz1 deposition [28], yet empirical evidence probing Arp6 function *in vivo* is very limited.

In addition to a role in chromatin modulating complexes, some nuclear ARPs are thought to have functions beyond that of the remodeling complex to which they belong. In other cases, such as Arp4 (BAF53 in mammals), ARP proteins are involved in multiple complexes, so that the phenotypes associated with *ARP* gene deletion are more extensive than those provoked by loss of a complex-specific ATPase subunit. Indeed, *ARP4* is essential for viability in yeast, and the protein is a component of both INO80- and SWR-C remodeling complexes and the NuA4 histone acetyltransferase, which carry out distinct nuclear functions [22], [39], [40]. Intriguingly, biochemical fractionation suggests that even these three complexes do not account for the entire nuclear complement of Arp4 [22], [39]. Other support for independent functions for ARPs comes from genome-wide screens for synthetic lethality. The 125 gene deletions that are lethal for cells lacking Arp6, for instance, are not necessarily lethal for cells lacking the Swr1 ATPase subunit [41]. Finally, the human Arp8 (hArp8) was implicated in mitotic chromosome phenotypes that could not be attributed to the hINO80 chromatin remodeling complex to which it belongs [42].

Here we have localized Arp6 along budding yeast chromosomes, both in the absence and presence of Swr1. We find that most Arp6 co-localizes with Swr1, being enriched in the promoters of divergently transcribed genes. This correlates with the deposition of the histone H2A variant H2A.Z/Htz1, and is consistent with the proposal that Arp6, as part of SWR-C, contributes to transcriptional regulation by exchanging H2A for Htz1 [33], [35]. Intriguingly, however, Arp6 binds some promoters in a Swr1-independent manner, including promoters of ribosomal protein genes. Indeed, transcript measurements show that Arp6 alters RP gene regulation independently of Swr1-mediated Htz1 deposition. We find that Arp6 can relocate chromatin to the NE independently of Swr1, and that *arp6* deletion reduces the association of RP genes with the NPC. This leads to a slight elevation in RP gene expression. We argue that Arp6 not only modulates local chromatin organization by facilitating Htz1 deposition, but also contributes to long-range chromatin organization that can fine-tune expression levels independently of SWR-C.

## Results

### Co-localization of Arp6 and Swr1 at yeast promoter regions

We determined the localization of Arp6 and Swr1 along budding yeast chromosomes by chromatin immunoprecipitation using high-density microarray chips (ChIP-chip assay). A 3FLAG epitope tag was introduced at the 3′ end of the genomic copy of either *ARP6* or *SWR1*, and the functionality of the fusion constructs was confirmed by monitoring growth rates and sensitivity to DNA damaging agents (Figure S1). Genomic DNA fragments (mean size ≅ 100 bp) were recovered, together with either Arp6 or Swr1 by anti-FLAG immunoprecipitation from formaldehyde-fixed and sonicated cells grown asynchronously in glucose-containing media (YPD). The fragments isolated by ChIP, as well as those in the total extract, were labeled with fluorescent dyes and hybridized to a high-density oligonucleotide array covering chromosomes 3, 4, 5, and 6R at either 100-bp or 300-bp resolution. With eleven 25-nucleotide probes for each 300-bp locus, the reliability of signal strength could be evaluated robustly by calculating the p-value for each locus (p<0.025) [43]. Reliability of the log2 ratio of the ChIP fraction recovery to the supernatant fraction was scored, and allowed discrimination of significant positive (yellow bars) and negative signals (open bars) for binding with overall resolution of ∼300 bp [43] (data available at www.ncbi.nlm.nih.gov/geo under the access number GSE9213).

**Figure 1:**
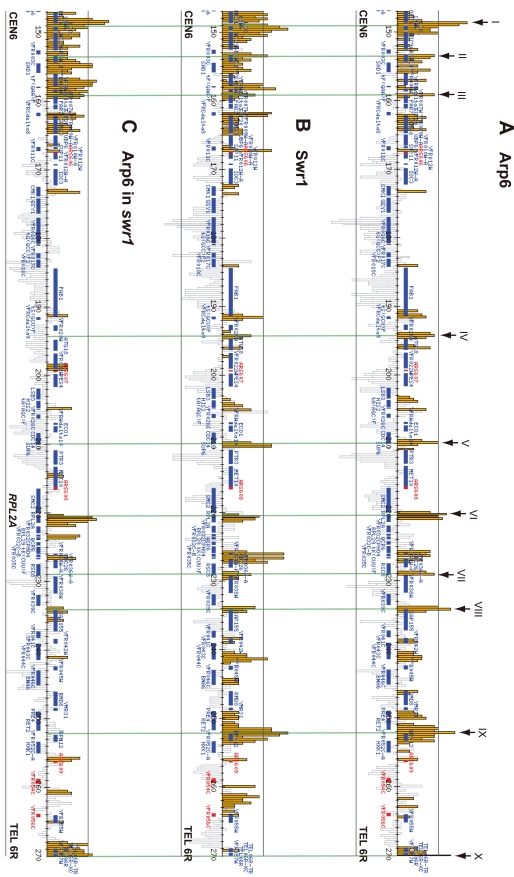
Localization of Arp6 and Swr1 on chromosome 6R. Vertical bars represent the binding ratio of proteins at each locus. Filled bars with yellow and black were determined to be significantly positive, for loci covering 300-bp or 100-bp regions, respectively. Open bars were not significantly positive. The scale of the vertical axis is log2 and the upper and lower horizontal lines represent 1.0 and -1.0, respectively. The central horizontal axis shows kilobase units. (A) Localization of Arp6-FLAG in *SWR1* wild-type cells. (B) Localization of Swr1-FLAG. (C) Localization of Arp6-FLAG in *swr1* cells. Vertical arrows (I to X) in A and vertical green lines represent the position of the highest ten clusters consisting of at least two continuous binding loci in both Arp6-and Swr1-FLAG ChIP.

Arp6 binding sites were found widely dispersed along chromosomes 3, 4, 5, and 6R, occasionally spreading over regions of several kb (Figures S2, S3, S5, and Figure 1, respectively). Among 8441 detectable chromosomal loci, 1498 loci were evaluated as positive for Arp6 binding (Table 1). Among the Arp6-binding sites, 71% also tested positive for Swr1, suggesting that Arp6 co-localizes with Swr1 at the majority of its chromosomal binding sites. The frequency of co-localization does not vary significantly among the chromosomes tested (Table 1). In Figure 1A, vertical arrows indicate the top ten peaks (signal log ratio >0.8) for Arp6 binding on Chr 6. Interestingly, one of the Arp6 peaks encompasses the centromere (arrow I), and a second is subtelomeric, containing unique sequences adjacent to the TG-rich repeats (arrow X). Arp6 binding sites generally coincided well with those of Swr1 (Figure 1A and 1B), except in subtelomeric zones where Arp6 binds alone (arrow X). Co-localization patterns were similar on all chromosome arms analysed (Figures S2, S3, S5). A closer examination of Arp6 promoter binding on Chr 6, indicates that Arp6 and Swr1 bind preferentially at the promoters of divergently transcribed genes (Figure S6, gray shade). These sites also contain Htz1 [35], and the coincidence of all three signals suggests that SWR-C stays bound after depositing Htz1 (H2A.Z), presumably to modulate promoter accessibility.

**Table 1:** Correlation of localization of Arp6 and Swr1 on budding yeast chromosomes.

		Chr3 (1322)	Chr4 (4857)	Chr5 (1849)	Chr6R (413)	Total (8441)
*SWR1*	Arp6 binding loci	383 (29%)	1117 (23%)	419 (23%)	102 (25%)	2021 (24%)
	coincidence with Swr1 binding	306 (80%)	817 (73%)	324 (77%)	81 (79%)	1528 (76%)
*swr1*	Arp6 binding loci	175 (13%)	671 (14%)	293(16%)	73 (18%)	1216 (14%)

In conclusion, our ChIP-chip analysis suggests that Arp6 binds chromatin as a component of SWR-C at most euchromatic sites, and in particular in the promoters of divergently transcribed genes. However, at centromeres, some telomeres and select promoters, Arp6 appears to bind independently of Swr1 (Figure 1C, Table S1, and below).

### Comparison of Arp6- and Swr1-containing complexes

**Figure 2:**
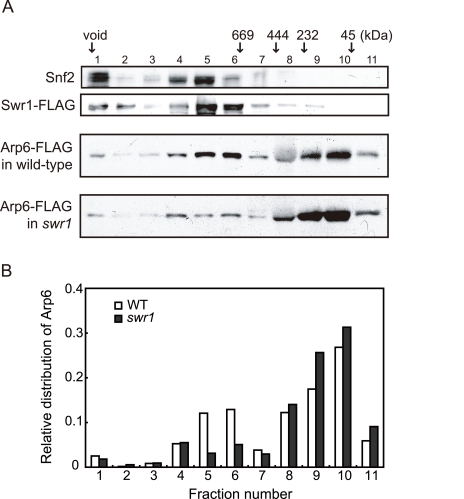
Sizing column Superose6 analysis of Arp6-complexes. (A) Extracts from cells expressing Arp6-FLAG under wild-type and *swr1* background were fractionated on a Superose6 column, and Arp6-FLAG in the fractions was detected on a Western blot with an anti-FLAG antibody (two bottom panels). The extract from cells expressing Swr1-FLAG was fractionated as well, and endogenous Snf2 and Swr1-FLAG were detected with an anti-Snf2 antibody and an anti-FLAG antibody, respectively (two top panels). The numbers of fractions and the positions of molecular mass standards are shown over the panels. (B) The intensity of Arp6-FLAG in the fractions were quantified, and relative distributions of Arp6 in the fractions of wild-type (WT) and *swr1* cells were shown.

The fact that Arp6 ChIP recovered the sites where Swr1 does not bind suggests that Arp6 may associate with other nuclear proteins or complexes, although to date it has only been reported to be a component of SWR-C. To test this possibility, we analyzed the native molecular masses of Swr1- and Arp6-containing complexes using gel filtration chromatography (Figure 2). Swr1 is recovered almost exclusively in fractions of ∼1–2 MDa (Figure 2A, second panel, lanes 5 and 6), and fractionates similarly to the catalytic ATPase of the SWI/SNF remodeler Snf2 (Figure 2A, first panel). In the presence of Swr1, Arp6 was distributed in both the 1–2 MDa Swr1-containing fractions, and in fractions that correspond to a molecular weight of 100–250 kDa (Figure 2A, third panel). In this latter fraction the complexes are still likely to be larger than the 57-kDa Arp6 monomer. Upon deletion of Swr1, the presence of Arp6 in the high MW fractions was significantly reduced (Figure 2A, fourth panel, and Figure 2B), and its abundance in the lower MW fractions increased. Our observations are consistent with the observation that Swr1 serves as platform for the assembly of SWR-C subunits, and that in the absence of Swr1, Arp6 only retains association with Swc6 [28], [44]. Nonetheless, a small amount of Arp6 was still recovered in a high MW complex in the *swr1*Δ strain (>1 MDa, Figure 2A, fourth panel). This may reflect participation of Arp6 in another large complex, albeit one of lower abundance. Quantitation of Arp6 recovery in both wild-type and *swr1*Δ cells, suggests that 30% of Arp6 is part of SWR-C or another large complex (Figure 2B), while the majority of Arp6 self-dimerizes or forms a complex with other small proteins. The nature of these is unknown, but the only reported partners of Arp6 in a *swr1*Δ strain are Swc6 and nucleosomes [28].

### SWR-C independent chromatin association of Arp6

**Figure 3:**
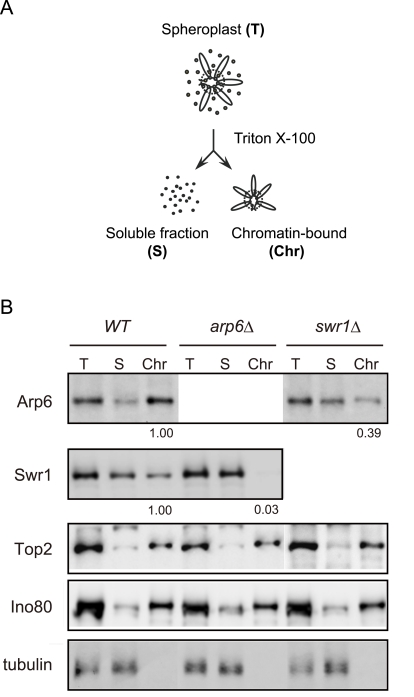
Arp6 partitions between soluble and insoluble chromatin fractions. (A) The fractionation protocol is shown. Yeast spheroplasts from appropriate strains were lysed with Triton-X100. A gentle centrifugation step separates a supernatant containing the bulk of cellular proteins from a chromatin pellet. (B) Wild-type, *swr1*, and *arp6* cells were subjected to the fractionation protocol described in (A), and the spheroplast (T), soluble fraction (S), and chromatin-bound (Chr) samples were probed using Western blot for Arp6-FLAG, Swr1-FLAG, topoisomerase II (Top2), the enzymatic component of the INO80 chromatin remodeling complexes (Ino80-MYC), and the soluble non-chromatin protein, tubulin. Numbers under panels show the ratios of chromatin-bound Arp6 and Swr1 in the mutants relative to WT. Their intensities were normalized with chromatin-preparation efficiencies obtained by quantification of the Western blot for Ino80.

To examine the ability of Arp6 to bind chromatin with or without Swr1, we fractionated yeast cells into a chromatin and a soluble protein fraction, using the well-established TritonX-100 lysis procedure [45]. This analysis confirmed that the majority of Arp6 is associated with chromatin in wild-type cells (Figure 3B). This is also true for tightly bound chromatin proteins like Ino80, topoisomerase II and ORC (Figure 3B and data not shown). Consistent with the finding that most Arp6-binding sites coincide with those of Swr1, we found that the association of Swr1 with chromatin required the presence of Arp6 (Figure 3B, *arp6*, lane Chr). This is unlikely to reflect an indirect effect on chromatin, since the association of Ino80 or topoisomerase II with chromatin was unaffected by *arp6* deletion (Figure 3B). In contrast, a large fraction of Arp6 (39% compared to wild-type) remained associated with chromatin even in the absence of Swr1 (Figure 3B, *swr1*, lane Chr). This result suggests that the physical association of Arp6 with chromatin is at least in part independent of SWR-C, and is consistent with the ChIP-chip data which show partially non-overlapping distributions of Arp6 and Swr1 (Figure 1).

### Detection of the Arp6 binding loci in the absence of Swr1

To elucidate the SWR-C-dependent and -independent functions of Arp6, we performed ChIP-chip analysis for Arp6 in a strain lacking *SWR1* entirely (*swr1*Δ). Consistent with the reduced level of chromatin-bound Arp6 (Figure 3B), fewer Arp6-binding sites were found in *swr1*Δ cells (Table 1). The major binding sites lost were those where both Arp6 and Swr1 colocalize in 5′ promoter regions (Figure 1, Figure S6, and Table 1), including the intergenic region 5′ of the *SWR1* gene itself (Figure S4).

To quantify the effect of *swr1* deletion on Arp6 binding patterns, we compared the log ratio of each Arp6-binding site in wild-type and *swr1*Δ cells (Figure S7A and S7B) with the log ratio of Swr1 binding at that locus. Generally, at the sites where both Arp6 and Swr1 were bound in wild-type cells, Arp6 binding was lost in *swr1*Δ cells (Figure 1 and Figures S2, S3, S5, S6). However, the overall distribution of Arp6 in the *swr1*Δ strain changes; notably, values increased at sites where Swr1 was not bound in the wild-type background (Figure S7B). This argues that in addition to an overall reduction in Arp6 binding, preferred Arp6 binding positions were altered in the absence of Swr1. This change in Arp6 binding suggests that SWR-C either competes for a limiting pool of Arp6 or alters chromatin such that some Arp6 binding sites are inaccessible, possibly reflecting indirect effects of Htz1 deposition.

Importantly, a subfraction of Arp6 binding sites persist in both wild-type and *swr1*Δ cells (see Figure 1). This argues, consistent with the fractionation data (Figure 3B), that Arp6 binds a subset of chromosomal loci independently of SWR-C, even in wild-type cells. Examples of this are RP gene promoters (e.g. *RPL2A*, Figure 1, arrow VI) and the Tel6R subtelomeric zone, which contains the inducible gene *HXK1* (Figure 1, arrow X). Indeed, Swr1-independent binding of Arp6 was enriched generally in a number of subtelomeric regions (Table S1). Despite the difficulty of analyzing subtelomeric domains on microarrays due to the presence of repetitive sequences, persistent Arp6 binding could be confirmed at Tel6R, Tel3L, Tel3R, and Tel4R in the absence of Swr1 (Figure 1, Figures S2, S3).

### Involvement of Arp6 in the expression of RP genes independently of H2A.Z-deposition

**Figure 4:**
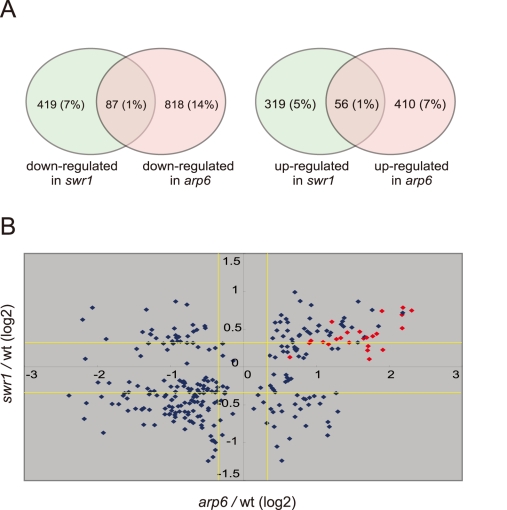
Transcript analysis in *swr1* and *arp6* mutant cells. Microarray analysis was repeated at least three times for *swr1Δ* and *arp6Δ* strain, and the statistical differences were determined using a t-test. p<0.05 was considered significant. (A) The Venn diagrams illustrate the degree of overlap between genes whose RNA levels were changed by 1.25-fold in *arp6* and *swr1* mutants. Numbers correspond to misregulated genes in the total genes. Relative numbers of the misregulated genes (%/total number of yeast gene) are indicated in parentheses. (B) Genes whose transcriptional changes were statistically significant (p<0.05) both in *arp6* and *swr1* cells were plotted according to their log2 ratios. The yellow lines show the 1.25-fold changes. The red diamonds represents RP genes.

To examine the independent contributions of Arp6 and Swr1 to gene expression, we performed a yeast whole-genome microarray with wild-type, *arp6*Δ, and *swr1*Δ cells (Table S2) (details available at www.ncbi.nlm.nih.gov/geo under the access number GSE17780). This expression microarray analysis was repeated at least three times for each strain, and the statistical differences were determined by t-test. Changes with p<0.05 were considered significant. We found a larger number of genes to be differentially regulated in both *arp6*Δ and *swr1*Δ cells (Figure 4A). When we compared the misregulated genes between the two mutants, we found that 87 out of 506 genes repressed in *swr1*Δ (see down-regulated in *swr1*, Figure 4A) are also down-regulated in *arp6*Δ cells (17% overlap), and 56 out of 375 genes induced in *swr1*Δ (see up-regulated in *swr1*, Figure 4A) are also up-regulated in *arp6*Δ cells (15% overlap; Figure 4A and Table S2). This overlap of down- or up-regulated genes between *swr1*Δ and *arp6*Δ strains was less than that reported for down- or up-regulated genes between *swr1* and *htz1* mutants (44% and 38%, respectively) [30]. Our expression data are consistent with our biochemical and ChIP analyses, which suggest that a majority of Arp6 is not recovered with SWR-C by sedimentation analysis (Figure 2B). The divergence of misregulated genes in the two mutants suggests that Arp6 can influence gene expression independently of SWR-C.

To extract more information on the differential effects of Arp6 and Swr1 in gene expression, we compared the degree of change in transcript level for each gene whose misregulation was significant (p<0.05) in both *arp6*Δ and *swr1*Δ strains (Figure 4B). Intriguingly, the degree of transcriptional change as a consequence of *arp6* deletion was greater than that provoked by *swr1* deletion. On the other hand, the deposition of Htz1 to promoters were similarly impaired in *arp6*Δ and *swr1*Δ cells, as previously reported [28], [29] (Figure S8). This analysis further indicates that Arp6 contributes to gene expression not only through Htz1 deposition, but also through a Swr1-independent mechanism.

**Figure 5:**
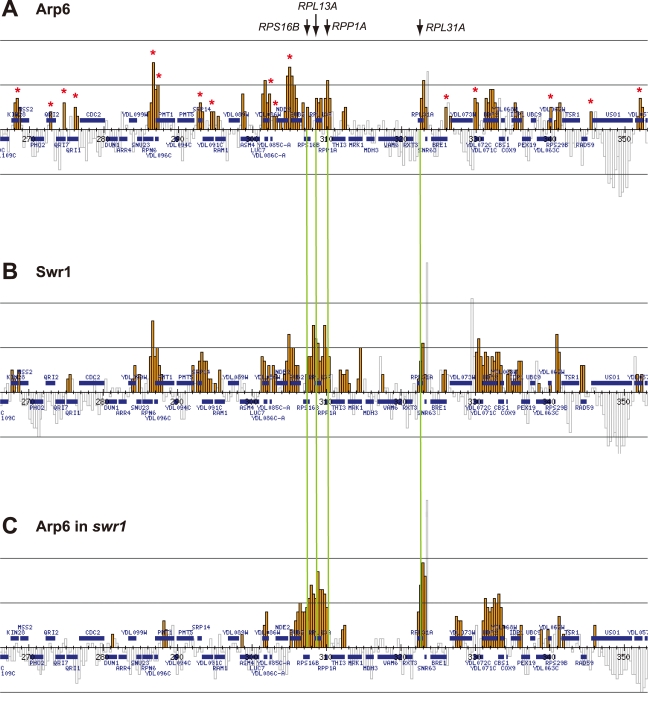
Swr1-independent binding of Arp6 to RP genes. Vertical bars represent the binding ratio of proteins in each locus. The binding of Arp6-FLAG (top), Swr1-FLAG (middle), and Arp6-FLAG in *swr1* cells (bottom) in the region 266K-353K of Chr 4L were compared. The positions of the RP genes (*RPS16B*, *RPL13A*, *RPP1A*, and *RPL31A*) in the region are shown with arrows and green lines. Red asterisks indicate those Arp6-gene promoter bindings which disappeared in the absence of Swr1.

Strikingly, among the 40 most up-regulated genes in *arp6*Δ cells we found 21 ribosomal protein genes (Table 2). Some of these RP genes were also modestly up-regulated in *swr1*Δ cells. Consistently, the ChIP-chip analysis in chromosomes 3, 4, 5, and 6R (Figures S2, S3, S5, and Figure 1, respectively) revealed that Arp6 and Swr1 bind to 25, and respectively 24, of the 27 RP genes on these chromosomes (Table S3). Importantly, and in contrast to most other euchromatic loci, Arp6 remained bound to all of these RP genes (including *RPS16B*, *RPL13A*, *RPP1A*, *RPL31A*, and *RPL2A*), even after deletion of *SWR1* (Figure 1, Figure 5, Figures S2, S3, S5, and Table S3). When we compared the transcription of RP genes between *arp6*Δ and *swr1*Δ cells, we find these genes more significantly up-regulated by loss of Arp6 than by loss of Swr1 (Figure 4B, red diamonds, and Table S4). From this we conclude that an Arp6-dependent, but Swr1- and Htz1-independent, mechanism modulates RP gene expression.

**Table 2:** Genes markedly up-regulated in *arp6* cells.

ORF name	*arp6*/wt log2 ratio	*swr1*/wt log2 ratio	Gene name	Description
YDL081C	2.31	0.75	RPP1A	Ribosomal protein
YOR167C	2.19	0.78	RPS28A	Ribosomal protein
YOR248W	2.19	0.72	TOS11	Questionable ORF
YGL030W	2.18	0.51	RPL30	Ribosomal subunit
YHR021C	2.18	0.69	RPS27B	Ribosomal protein
YHR143W-A	2.03	(0.03)	RPC10	Subunit of RNA polymerase II
YGL147C	2.00	(0.06)	RPL9A	Ribosomal protein
YDL083C	1.91	0.22	RPS16B	Ribosomal protein
YJR123W	1.90	0.74	RPS5	Ribosomal protein
YOL014W	1.89	(0.32)		Hypothetical protein
YPR078C	1.85	0.54		Hypothetical protein
YLR185W	1.83	(0.45)	RPL37A	Ribosomal protein
YDL130W	1.83	0.44	RPP1B	Ribosomal protein
YJR145C	1.77	0.41	RPS4A	Ribosomal protein
YPL163C	1.77	0.75	SVS1	Serine- and threonine-rich protein
YAL025C	1.74	(−0.50)	MAK16	Putative nuclear protein
YLR264W	1.73	0.10	RPS28B	Ribosomal protein
YNL162W	1.73	(0.08)	RPL42A	Ribosomal protein
YKL006W	1.70	0.28	RPL14A	Ribosomal protein
YHL001W	1.70	0.22	RPL14B	Ribosomal protein
YBL087C	1.69	0.39	RPL23A	Ribosomal protein
YBL071C	1.67	0.40		Hypothetical protein
YPL220W	1.65	0.38	RPL1A	Ribosomal protein
YBR267W	1.63	(−0.22)	REI1	Cytoplasmic pre-60S factor
YGL135W	1.60	0.47	RPL1B	Ribosomal protein
YNL333W	1.59	(0.24)	SNZ2	Member of the stationary phase-induced gene family
YHR072W-A	1.58	(0.19)	NOP10	Component of H/ACA-box snoRNPs
YLR075W	1.58	(0.11)	RPL10	Ribosomal protein
YOR292C	1.57	0.86		Hypothetical protein
YAL012W	1.57	(0.52)	CYS3	Cystathionine gamma-lyase
YAR009C	1.57	0.32	TY1B	Ty1B protein
YDL082W	1.55	(−0.10)	RPL13A	Ribosomal protein
YDR101C	1.53	(−0.22)	ARX1	Shuttling pre-60S factor
YNL255C	1.53	(0.36)	GIS2	Contains seven cysteine-rich zinc finger motifs
YPL093W	1.53	(−0.04)	NOG1	Nucleolar G-protein (putative)
YDR184C	1.51	(−0.21)	ATC1	Nuclear protein that interacts with Aip3
YOR096W	1.51	(−0.14)	RPS7A	Ribosomal protein
YJL136C	1.51	(0.19)	RPS21B	Ribosomal protein
YLR110C	1.49	(1.03)	CCW12	Cell wall mannoprotein
YLR157C-B	1.48	(0.52)		Transposable element gene

Parenthesis: change is not significant (p>0.05).

**Figure 6:**
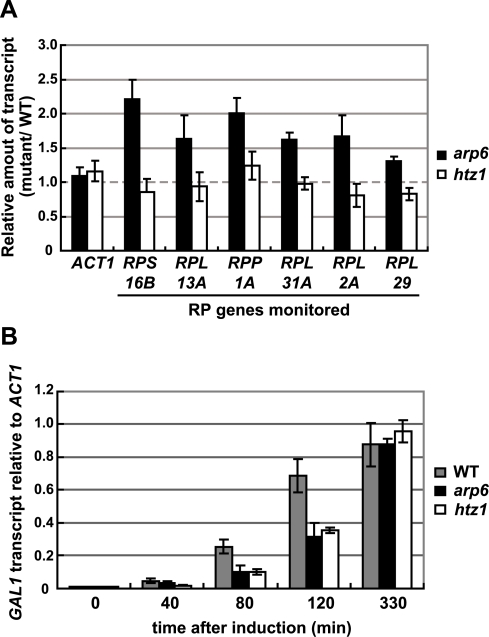
Quantitative analysis of transcripts in cells lacking Arp6 (*arp6*) and H2A.Z *(htz1*) using RT–PCR. (A) The same amount of total RNA from wild-type, *arp6*, and *htz1* cells was analyzed using quantitative RT–PCR by using primer sets specific for each of the RP genes. The *ACT1* gene was analyzed as a control. The relative amount of the transcript of the genes in *arp6* and *htz1* to wild-type is shown by filled and open bars, respectively. The data points represent the mean ± SD for at least three experiments. (B) Total RNA was prepared from wild-type, *arp6*, and *htz1* cells (gray, filled, and open bars, respectively) at the indicated times after induction of *GAL1* expression in galactose. The amounts of *GAL1* transcript in these strains were analyzed using quantitative RT–PCR, and shown as relative amounts compared to *ACT1* transcript. The data points represent the mean ± SD for at least three experiments.

Consistent with our analysis, it was reported earlier that Htz1 is excluded from RP genes [34], [35]. Moreover, microarray analyses have shown that the absence of Htz1 does not have any significant effects on RP gene expression [30], [37], [46]. To confirm this, we examined the expression of the relevant RP genes by quantitative rtPCR. We could confirm that transcript levels were not significantly altered by loss of Htz1, yet were increased in *arp6*Δ (Figure 6A). In contrast, other Arp6-bound promoters that are known to be regulated by SWR-C mediated deposition of Htz1 (e.g. *GAL1* [31], [47]) showed a reduction or delay in induction by galactose that was similar in both *htz1*Δ and *arp6*Δ cells (Figure 6B). This could be extended to several non-inducible genes, to which Arp6 binds in Swr1-dependent manner such as *RDS1* (YCR106W) and *UBX3* (YDL091C) (Figure S9). These genes, like the inducible *GAL1*, showed a similar decrease in expression in both *htz1*Δ and *arp6*Δ cells (Figure S9, filled and gray bars, respectively). Our observations argue that Arp6 is involved in gene expression in both the Htz1-dependent and Htz1-independent pathways. Moreover, Arp6 binding can both increase and lower transcript levels: at *GAL1*, where Htz1 is deposited in an Arp6- and Swr1-dependent manner, expression is less efficient in the absence of Arp6 or Htz1 deposition, while at RP genes, where Arp6 binding is independent of Swr1 and Htz1, its absence increases expression levels.

### Arp6 mediates relocalization of chromatin in a Swr1-independent manner

**Figure 7:**
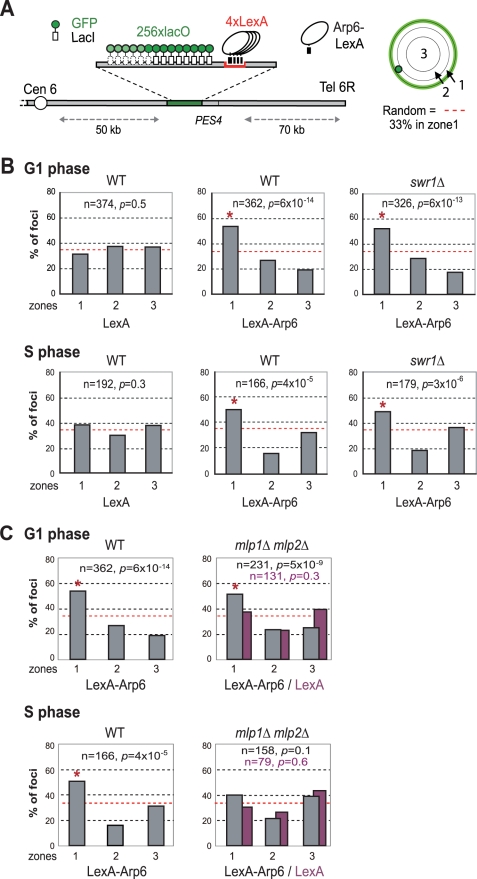
Perinuclear anchoring activity of Arp6. (A) The ability of a LexA fusion to relocate the lacO-tagged *PES4* locus bearing LexA binding sites was tested using a strain GA-1461. *PES4* is located 70 kb and 50 kb from Tel6R and Cen6, respectively. The lacO array was visualized by binding a GFP-LacI fusion, and the nuclear envelope is visualized through a Nup49-GFP fusion. The focal plane in which the GFP spot was brightest was used to monitor distances, which were reported as a ratio to nuclear diameter. These values were binned into one of three concentric zones of equal surface and are presented as percentage of total spots scored. The position of *PES4* was mapped in wild-type (WT) and *swr1* cells expressing LexA alone or a LexA-Arp6 fusion. (B) Arp6 can relocalize an internal chromatin locus (*PES4)* to the nuclear periphery. Cells were classified as G1 (unbudded) or S phase (budded with spherical nucleus). (C) Mlp1/Mlp2 is required for the perinuclear anchoring by Arp6 in S-phase cells. The position of the *lacO* arrays was scored on *PES4::lacO* tagged cells expressing LexA (purple) or LexA-Arp6 (grey). The bar graphs show the percentage of spots (y-axis) per zone (x-axis) for G1-phase (upper panels) and S-phase (lower panels) cells. The number of cells analyzed (n) and the confidence values (p) for the χ^2^ analysis between random and test distributions are indicated. The red dashed horizontal line at 33% indicates a random distribution, and zone 1 distributions that are significantly different from random are indicated by an asterisk (p<0.05).

Recent studies have suggested that not only local changes in chromatin organization, but also long-range chromatin organization can influence gene expression. Genome-wide ChIP-chip analysis for nuclear pore components has shown that RP genes associate with components of the NPC [9]. Given that Arp6 associates with most RP genes in the absence of Swr1, we wondered whether the Arp6-specific effect on transcription might be mediated through an interaction of the target gene with nuclear pores. To examine this possibility, we made use of an assay that scores for the ability of a protein fused to LexA to shift a randomly positioned chromosomal locus to the nuclear periphery [2]. This assay has been used to identify protein domains that are sufficient for interaction with structural components of the NE. The locus chosen is a constitutively expressed gene near ARS607 (*PES4)* at which we have inserted 4 LexA binding sites and a lac operator array that allows visualization with GFP-LacI (Figure 7A) [2]. Proteins that are to be tested for perinuclear relocalization activity are expressed as LexA fusions in a strain expressing GFP-Nup49 to tag the NE. Unlike the expression of LexA alone (Figure 7B, LexA), the LexA-Arp6 fusion protein led to an enrichment of the *PES4* locus in the outermost nuclear zone (zone 1) in both G1- and S-phase cells (Figure 7B, LexA-Arp6 in WT). Importantly, the relocalization activity of LexA-Arp6 was independent of Swr1 (Figure 7B, LexA-Arp6 in *swr1*). Expression of LexA alone does not shift the position of the tagged locus, allowing us to conclude that LexA targeted Arp6 is sufficient to favor the association of a chromatin locus with the NE. This is independent of Htz1 deposition, since it requires the catalytic activity of Swr1. We note that there is a low level of endogenous Arp6 detected near the *PES4* locus in *swr1* mutant cells (Figure 1), yet this is insufficient to tether a significant fraction of the sites to the NE (see LexA alone).

### Arp6 is required for the association of chromatin with the NPC

**Figure 8:**
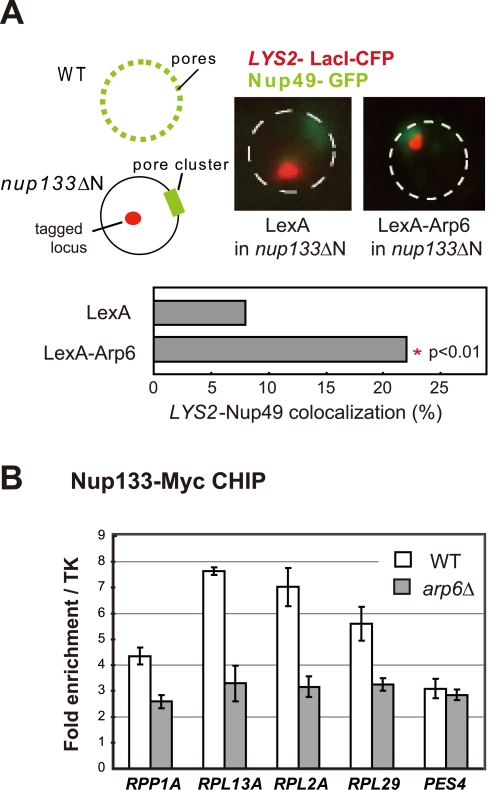
Involvement of Arp6 in intranuclear organization through the NPC. (A) The positions of *lacO*-tagged *LYS2* (red) and of CFP-Nup49 (green) were observed in a *nup133ΔN* background, in which nuclear pores cluster on one side of the nucleus. Bar graphs represent the percentage of complete red-green signal overlap counted in cells expressing LexA alone or a LexA-Arp6 fusion. The confidence values (p) for the χ^2^ analysis between them is indicated. The predicted colocalization for a randomly positioned locus is 9% [49]. (B) Requirement of Arp6 for the interaction of RP genes with the NPC. The association of Nup133-Myc with RP genes, *RPP1A*, *RPL13A*, *RPL2A*, and *RPL29*, was quantified using ChIP analysis combined with quantitative PCR in wild-type (WT) and *arp6* cells, and is plotted over a background control of the TK gene [80]. The *PES4* locus was analyzed as a control. The data points represent the mean ± SD for at least three experiments.

To confirm that the Arp6-bound locus associates with nuclear pores, as opposed to other perinuclear sites, we performed the relocalization assay in a strain that expresses a nuclear pore protein Nup133 that lacks its N-terminal domain (*nup133*ΔN) [48]. In this mutant, functional NPCs cluster on one side of the nucleus allowing us to monitor whether a LexA-Arp6 targeted locus moves to pores or to other sites on the nuclear envelope (Figure 8A). Compared to cells expressing LexA alone, the *LYS2* locus bound by LexA-Arp6 not only accumulated in the nuclear peripheral zone like the *PES4* locus (49% in zone 1 vs 34% for LexA alone in G1-phase cells, data not shown) but also colocalized significantly with clustered NPC (Figure 8A; 22.4%, n = 322, p<0.01). Previous studies have shown that a randomly distributed tagged locus would coincide with a pore cluster in 9% of the cells, while a locus that has a predisposition to be perinuclear (i.e. 60% occupation of zone 1) would coincide with a pore cluster in 10% of cell scored [49]. The 22% scored for Arp6 relocation versus the 8% scored for the control LexA is thus highly significant. It is comparable to the ∼two-fold increase in colocalization achieved by targeting LexA-Nup84 vs LexA alone [49]. This rate of colocalization suggests that a component of the NPC is able to bind Arp6.

We next used quantitative ChIP analysis to test whether the loss of Arp6 influences the association of endogenous RP genes with the NPC. Immunoprecipitation of Nup133-Myc confirmed that the RP genes tested previously [9] are associated with pores and that deletion of *arp6* reduces the recovery of these genes with Nup133 (Figure 8B). *PES4,* a randomly positioned locus with no natural affinity for nuclear pores, did not precipitate significantly with Nup133 and was unaffected by *arp6* deletion (Figure 8B). From this we conclude that Arp6 is required for the RP gene-NPC interaction (Figure 8B).

**Figure 9:**
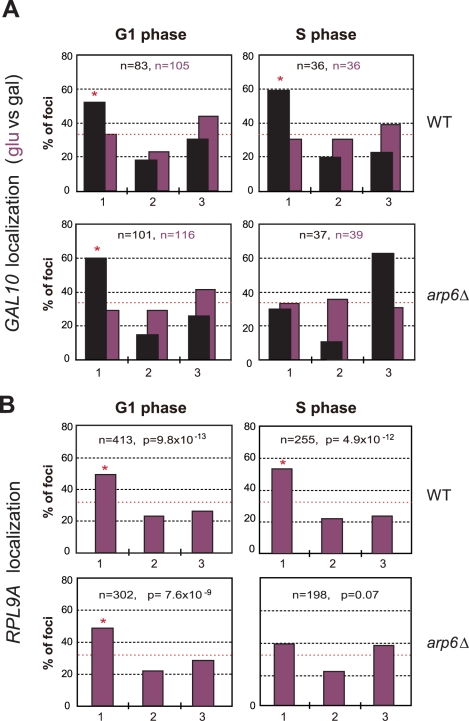
Arp6 is required for the peripheral association of galactose-induced *GAL10* and constitutively expressed RP gene *RPL9A*. (A) The *GAL1-GAL10* locus was tagged by inserting 256 *lac* operators in a haploid wild-type or *arp6* deletion strain bearing GFP-lacI and Nup49-GFP fusions (wild-type; GA-4098, *arp6*; GA-6024) [81]. The position of the *lacO* arrays relative to the nuclear envelope was scored on images take of living cells growth either on glucose (purple) or after 2 hours of gene induction on 2% galactose (black). Three zone scoring was carried out as in Figure 7. The number of cells analyzed for each stage of the cell cycle are indicated, and the confidence values (p) for the χ^2^ analysis between random and test distributions on galactose are: wild-type (G1, p = 4×10^−4^; S, p = 6×10^−4^) and *arp6* (G1, p = 2.7×10^−8^; S, p = 0.44) none of the values on glucose are significantly different from random (p>0.05). The G1-S differences on galactose are not significant in WT, but are in the *arp6* mutant (p = 0.024). Note that in this analysis we omitted the rare, very small budded cells. (B) The *RPL9A* locus was tagged by inserting 256 *lac* operators in a haploid wild-type or *arp6* deletion strain bearing GFP-lacI and Nup49-GFP fusions (wild-type; GA-3635, *arp6*; GA-5132) [81]. The position of the *lacO* arrays relative to the nuclear envelope was scored as in Figure 7 on image stacks taken on living cells grown on SC. Symbols and quantitation are as in A. *RPL9A* locus p values for test vs random distributions are: wild-type (G1, p = 9.8×10^−13^; S, p = 4.9×10^−12^) and *arp6* (G1, p = 7.6×10^−9^; S, p = 0.07). The G1 vs S distributions in wild-type are not significantly different (p = 0.33) while in *arp6* cells the difference is significant (p = 0.028). Whereas values for Zone 1 in wild-type vs *arp6* cells in G1 are not significantly different (p = 0.82), the difference in mid-to-late S phase cells is (p = 0.0018). An asterisk indicates that values that have a nonrandom distribution (p<0.05). The number of cells analyzed (n) and the confidence values (p) for the χ^2^ analysis between random and test distributions are indicated.

We asked whether Nup133 was the only site of interaction for these genes with the nuclear envelope. In other words, we checked by lacO-tagging and scoring of subnuclear position, whether RP or *GAL1* genes would lose all perinuclear localization in absence of Arp6. We found that the galactose-induced relocalization of *GAL10* (which shares the *GAL1* promoter) to the NE was indeed lost in S-phase *arp6*Δ cells (Figure 9A), as was the constitutive association of the RP gene *RPL9*A (Figure 9B). Inexplicably, however, the loss of association provoked by *arp6* deletion was cell-cycle stage specific, arguing that an alternative, possibly redundant mechanism allowed loci to remain peripheral, although probably not associated with Nup133, in G1-phase cells. The effect was also at least partially locus- or context-specific, since a second tagged RP gene cluster at *RPP1A* was enriched at the nuclear periphery in both wild-type and *arp6*Δ strains (data not shown). Taken together our data argue that Arp6, while being sufficient to relocate loci to the NE (Figure 7), is not the only pathway that tethers active genes at nuclear pores. This was already suggested from the results from the Rosbash, Silver, Stutz, Hurt, Nehrbass, Brickner and Proudfoot laboratories, who have identified both SAGA-dependent and SAGA-independent pathways for locating active loci at nuclear pores [4]. The fact that *GAL10* and *RPL9A* association was ablated in S-phase by *arp6* deletion, suggests that a redundant pathway of anchoring functions primarily in G1 phase. Although it is unclear why transcription-regulated association with the NE, should be cell-cycle controlled, this is highly reminiscent of the distinct G1- and S-phase specific tethering mechanisms that mediate anchoring of telomeres [2], [50] and DNA damage [49].

We next examined whether other pore-associated proteins, namely, Mlp1 and Mlp2, myosin-like proteins associated with the inner nuclear basket, are involved in either the Arp6-mediated pathway of gene anchoring. They were likely candidates due to their implication in the association of *GAL10* and *HSP104* with nuclear pores, through Mex67 and Yra1 [51]–[53]. To test this, the position of the tagged *PES4* locus bound by LexA-Arp6 or by LexA alone was determined in strains carrying *mlp1Δ mlp2Δ* deletions (Figure 7C, *mlp1Δ mlp2Δ*). Intriguingly, we again see that LexA-Arp6 anchoring activity was dependent on Mlp1 and Mlp2 exclusively in S-phase cells. We conclude that Arp6 is able to mediate association with nuclear pores in an Mlp1/Mlp2-dependent manner, yet again our data indicate that a second pathway for Arp6 binding is functional in G1 phase. Arp6 may interact with the coiled-coil proteins Mlp1 and Mlp2 directly, although it is more likely to bind through Yra1 (see Discussion). Overall, our results support the notion that Arp6 has a role both in local chromatin modulation through H2A.Z-deposition, and in long-range chromatin organization through its ability to bind proteins associated with the NPC; an interaction which depends at least partially on the myosin-like proteins 1 and 2.

## Discussion

### SWR-C–dependent and–independent binding of Arp6 to chromatin

Our high resolution ChIP-chip assay has shown that Arp6 co-localizes with Swr1 at most of its euchromatic sites, presumably as a component of SWR-C [28], [30]. No conserved sequence motif for the binding of Arp6 or Swr1 could be identified (data not shown), partly because the targeted regions are relatively large and transcription factor binding sites are often degenerate. Moreover, it is possible that Arp6 is targeted by recognition of a specifically modified nucleosome and not a DNA binding factor [28]. Interestingly, the highest Arp6 occupancy was detected within a 300-bp fragment containing the start ATG codon of the *SWR1* gene. This coincides with a peak of Swr1 and argues for an auto-regulatory loop for *SWR1* expression (Figure S4) [35].

More generally, the ChIP-chip assay showed a coordinated enrichment for both Arp6 and Swr1 in intergenic regions particularly near the 5′ ends of divergently transcribed genes (Figure 1A and 1B). Of the top ten loci for Arp6 binding on Chr 6 (arrows, Figure 1A), five contain the start ATG codon of genes, two are located within 200 bp of an ATG codon, and two others are within 400 bp of an ATG codon. Only at telomeres are Arp6 sites more than 1 kb from the nearest ATG. The localization of Htz1 on yeast chromosomes has been previously examined in detail, and was shown to be present at the 5′ ends of most genes [33]–[35]. This suggests that Arp6 and Swr1 remain chromatin-bound at sites where they incorporate Htz1.

Unexpectedly, we also detected numerous loci that were positive for Arp6 but negative for Swr1 interaction (Figure 1A and 1B, Table 1). A large fraction of Arp6 was shown not be integrated in the SWR-C complex by gel filtration analysis. Moreover, although Swr1 association with chromatin was dependent on Arp6, about 40% of total Arp6 remained chromatin-bound in the absence of Swr1 (Figure 2 and Figure 3). Swr1 contributes not only to the catalytic activity of SWR-C, but also provides critical protein-protein contacts that maintain the integrity of the holocomplex [28]. Importantly, in a *swr1* deletion strain Swc2, the Htz1-binding module of SWR-C, dissociates from Arp6 and only Swc6 remains Arp6 bound [28]. Consistently, sites that bind Arp6 in the absence of Swr1 are anti-correlated with the presence of Htz1.

ChIP-chip analysis showed that the SWR-C-independent binding of Arp6 is observed at RP genes and in some subtelomeric zones, which are both depleted for Htz1 [37], [38], [54]. Intriguingly, we have also found that the perinuclear tethering of Tel6R is impaired in *arp6*, but not in *swr1* mutants (K.S., A.T. and S.M.G. unpublished data). The role of Arp6 at telomeres is therefore not restricted to the deposition of Htz1.

### Binding of Arp6 to RP genes

The RP genes are among the most important genes for cell metabolism, and the fine-tuning of RP gene transcription responds to a variety of environmental effects, ultimately coordinated by the TORC1 complex (Target of Rapamycin) [reviewed in 55]. However, exactly how these crucial genes are regulated at a transcriptional level is unclear. Several transcription factors, including Rap1, Fhl1, and the high mobility group protein, Hmo1, have been shown to play roles in the expression of RP genes. Previous studies suggested that Rap1 may recruit Fhl1 and Hmo1 to RP promoters [56], [57]. As shown here, Arp6 binds to most of the RP genes present on the chromosomes we analyzed. Given that Rap1 has significant genetic interactions with Arp6 [58], we speculate that Arp6 may cooperate with Rap1 to regulate the association of other factors to RP gene promoters. A particular constellation of factors may also contribute to the association of these genes with nuclear pores.

Kasahara et al. [59] compared the binding of Hmo1 with those of Fhl1 and Rap1 to RP genes using ChIP-chip analysis, and divided RP genes into classes that have either Hmo1-dependent or Hmo1-independent binding of Fhl1 and Rap1. We find no correlation of either class with the presence of Arp6 (data not shown). Moreover, Arp6 is bound at the promoters of *RPP1A*, *RPL4B*, and *RPP2B*, which belong to a subgroup that binds neither Hmo1, Fhl1, nor Rap1 [59]. Thus, while it is possible that Arp6 influences the binding of these factors, the converse is not true. We also note that, unlike loss of Hmo1, Fhl1 or Rap1, the absence of Arp6 leads to an increase in the expression level of genes such as *RPP1A* (Table 2). This argues that the binding of Arp6 reduces rather than enhances RP gene expression. It is important to note that RP genes are highly expressed, and therefore even a 50% drop in expression means that the gene is still actively transcribed. Thus the localization of RP genes to pores by Arp6 binding reduces but does not eliminate expression. This is not the first report of pore association leading to reduced expression: a gene in the heat-shock family, *HSP104*, which is associated with the NPC by an mRNA- and Mlp1/Mlp2-dependent pathway, also had higher expression levels when its association with the NPC was impaired [51].

### Arp6 is required for gene expression in a H2A.Z-dependent and -independent mechanism

We show here that the loss of Arp6 increases expression of RP genes by 1.5- to 2-fold, in a manner independent of Htz1 and SWR-C (Figure 4, Figure 6A, and Table 2). Arp6 binds many of these RP gene promoters and is required for their tight association with the nuclear pore protein Nup133 (Figure 8B). Given that chromatin-bound Arp6 can relocate genes to pores, we can conclude that Arp6 either directly or indirectly mediates the association of RP genes with pores. In general, the RP and non-RP genes that are most activated by *arp6* deletion (Table 2, e.g. YOR248W) are among those associated with NPCs [9]. This establishes for the first time a strong correlation between association with the NPC and down-regulation for a class of coordinately regulated genes.

There have been several reports showing that genes induced by non-glucose carbon sources, inositol starvation or heat shock associate with the NPC for optimal induction [5]–[7], [9], [60]. We confirm here that the association of *GAL1* with the NPC is Htz1- and Arp6- dependent [61] (Figures S8, S10), and that in the absence of either factor, induction occurs less rapidly, although the final mRNA level is unchanged (Figure 6B). Since Arp6 is required for H2A.Z-deposition, this result is not surprising. Still, it is important to contrast this result with that observed for RP genes: at *GAL1*, Arp6 contributes to gene activation in an Htz1- and Swr1-dependent manner, while at RP genes it contributes to down-regulation in an Htz1- and Swr1-independent manner. Both Arp6-mediated activities correlate with localization to the nuclear pore.

Our data extend and support previous studies that show that final mRNA levels from galactose- and stress-induced genes decrease if association with pores is impaired [6], [7], [61]. We find that among the 40 loci most significantly down-regulated by loss of Arp6, ten are heat-shock or stress-induced genes (Table S5). We confirm by ChIP-chip that Arp6 also binds these genes, albeit less avidly than it binds RP promoters (Figures S2, S3, S5). Since our analyses were not done under conditions of stress, we propose that basal level expression is also affected by Arp6-mediated Htz1 deposition. The induction of these genes often requires the SAGA histone acetyltransferase complex which further contributes to NPC tethering through Sus1 [17], complementing the Htz1 contact [61], [62]. Thus our results confirm that Arp6-pore association acts on two pathways relevant to a number of genes: binding through SWR-C and Htz1 deposition facilitates expression of stress-induced genes, and binding independently of Htz1 at RP promoters leads to a 1.5 to 2-fold down-regulation. This allows us to propose that nuclear pores are platforms for fine-tuning gene expression and not necessarily for enhancing initiation. The control steps may involve RNA processing, export or even RNA Pol II elongation.

Two essential mRNA export proteins, Mex67 and Yra1, have been implicated in NPC-gene association and gene expression [4], [51], [52]. Mex67 and Yra1 physically interact with Mlp1/Mlp2 proteins [52], [53], [63], [64], which we show here to be required for Arp6-mediated relocation to the NE in S-phase cells (Figure 7C). Interestingly, proteome analysis has identified Yra1 as a binding partner of Arp6, but not of Swr1 or Htz1 [65]. This raises the possibility that the Mlp-Yra1-Arp6 interaction allows for the perinuclear tethering of Arp6-bound RP genes (Figure 7). A redundant mechanism in G1 phase cells may account for the fact that Arp6 continues to anchor in this phase of the cell cycle in the *mlp1 mlp2* mutant.

Pore association may fine-tune RP gene expression through feed-back mechanisms that are driven by ribosomal protein levels [66]–[69]. If the loss of association of RP genes with the NPC in *arp6* cells initially reduces mRNA processing and export [70], then ensuing reduction in levels of ribosomal proteins themselves may feed-back to counteract repression, enhancing RP gene expression [66]. Consistently, the *RPS14B/CRY2* transcript levels are increased in cells defective in mRNA transport [68].

### Arp6 and long-range chromatin organization

In addition to facilitating mRNA processing and export, the association of euchromatic domains with the NPC may facilitate the formation of nuclear subcompartments by creating boundaries [6] or by recruiting proteins required for genetic function or epigenetic control [reviewed in 4]. We note that a large fraction of Arp6 is chromatin-bound even in the absence of Swr1 (Figure 3), and that 25% of total Arp6 can be recovered in a nuclease-resistant nuclear scaffold fraction (data not shown). The association of Arp6 with an insoluble fraction of the nucleus, together with its ability to influence the localization of genes, argue that Arp6 can contribute to long-range organization of chromatin in the interphase nucleus. The ability of Arp6 to relocate chromatin to pores is not characteristic of all Arp proteins; the targeting of Arp5, a component of the INO80 chromatin remodeling complex with related molecular properties, does not change the random distribution of the tagged *PES4* locus (H. van Attikum and S.M.G., personal communication). The perinuclear binding activity may thus reflect a unique domain of Arp6 or a binding partner with affinity for the NPC.

The positioning of chromatin in the interphase nucleus not only influences transcription, mRNA processing and export, but genome stability as well. Several laboratories have reported that critically short telomeres, irreparable DNA double-strand breaks and collapsed replication forks shift to the NPC for a repair pathway controlled by SUMO-dependent ubiquitin ligase [71]–[74]. Since *arp6* mutants show hypersensitivity to various DNA damaging agents, Arp6 may also contribute to repair through its perinuclear relocalization activity.

We have recently analyzed chicken DT40 cells carrying a conditional knockout for Arp6, and found that the radial distribution of chromosome territories was altered in the absence of Arp6 (Ohfuchi et al., submitted). We therefore entertain the hypothesis that the contribution of Arp6 to long-range chromatin organization is evolutionarily conserved. In vertebrates there is as yet no compelling data implicating the NPC in gene expression or DNA repair, although other intranuclear structures such as PML bodies or transcription factories may replace pores in this function. We note that the reduction of human Arp4 by siRNA, unlike the loss of BRG-1, BRM, or Tip49, causes an expansion of the nuclear volume occupied by individual chromosomes (chromosome territories, [75]). While the mechanism remains obscure, this is consistent with the proposal that ARPs have roles in the long-range organization of chromatin that are independent of chromatin remodeling activities. The challenge remains to understand how cells regulate the interaction of chromatin with ARPs, nuclear actin, myosin and known structural proteins like lamins and nuclear pores.

## Materials and Methods

### Plasmids, strains, and yeast imaging methods

The LexA-Arp6 fusion was constructed as in Taddei et al. [7]. Yeast transformations were done using the lithium acetate procedure, and PCR-based gene deletions and tagging were performed as described [76]. The genotypes of all strains used in this study are listed in Table S6. Standard culture conditions at 30°C were used unless otherwise indicated. A 6His-3FLAG tag was fused to the C-terminus of Arp6 or Swr1 using the cassette amplified from pU6H3FLAG (gift from Dr. De Antoni) [43]. Live fluorescence microscopy and quantification was performed according to Hediger et al. [77] and Taddei et al. [2].

### Chromatin immunoprecipitation (ChIP)–chip analysis

A chromosome III, IV, V, and VI right-arm high-density oligonucleotide chip was produced by Affymetrix Custom Express Service (SC3456a520015F, P/N, Affymetrix). Sequence and position of oligonucleotides on the microarrays are available from Affymetrix. ChIP was carried out as previously described [43] with a few modifications. Yeast cells were grown in 200 ml YPD medium for 12 hr at 30°C, cross-linked, and disrupted using a multi-beads shocker (MB400C, Yasui Kikai), which was able to keep cells precisely at lower than 6°C during disruption by Zr beads. The anti-FLAG monoclonal antibody M2 (Sigma-Aldrich) was used for ChIP. ChIP DNA was purified and amplified by random priming as previously described [78]. The total of amplified DNA was digested with DNaseI to a mean size of 100 bp, purified, and the fragments were end-labeled with biotin-N6-ddATP. Hybridization, washing, staining, and scanning were performed according to the manufacturer's instructions (Affymetrix). Data analyses were carried out as described previously [43].

### Microarray analysis

For microarray analysis, total RNAs were prepared from cultures grown at 30°C in YPD medium to OD_600_ = 1.0 using TRIzol (Invitrogen). Microarray detection was performed as previously described [79], and carried out on at least three independent cultures.

### Gel filtration analysis

The native molecular mass of complexes was monitored by gel filtration analysis according to Harata et al. [22] with modifications. Yeast extract from 100-ml culture of log-phase cells were applied to a Superose 6 column, and proteins were eluted at a flow rate of 0.2 ml/min. 1-ml fractions were collected and subjected to Western blot with the anti-FLAG M2 antibody to detect Arp6-Flag and Swr1-Flag. Snf2 was detected by using an anti-Snf2 antibody (Upstate).

### Chromatin fractionation assay

The chromatin fractionation assay was performed as previously described [45] with the following modification. After spheroplasting, cells ware washed twice in 50 mM Hepes-KOH pH 7.5, 20 mM KCl, 2 mM EDTA-KOH, 0.05 mM spermine, 0.125 mM spermidine, 1 M sorbitol, 1% Trasylol, and 1 mM PMSF. The pellet of spheroplasts (∼4×10^8^cells) was then re-suspended in 1 ml 50 mM Hepes-KOH pH 7.5, 2.5 mM MgCl_2_, 10 mM glycerol 2-phosphate, 0.1 mM Na_3_VO_4,_ 0.25% Triton X-100, 300 µg/ml benzamidine, 1 µg/ml pepstatin A, 2 µg/ml antipain, 0.5 µg/ml leupeptin, 100 µg/ml TPCK, 50 µg/ml TLCK.

### Quantitative PCR and ChIP analysis

For quantitative RT-PCR analysis, total RNAs were prepared from cultures grown at 30°C in YPD or YPG medium to OD_600_ = 1.0 by using RNeasy Mini Kit (Qiagen). Total RNAs were reverse-transcribed using the High Capacity cDNA Reverse Transcription kit (ABI), and subjected to quantitative real-time PCR with a SYBR Green Master Mix (ABI Prism 7000 Sequence Detector System and Software). ChIP was performed as for ChIP-chip analysis, but purified ChIP DNA was subjected to quantitative real-time PCR rather than microarray hybridization. For the primer sets, see Text S1. Real-time PCR monitors the threshold cycle at which the exponential curve of the accumulated product passes a threshold. PCR reactions were performed at least three times. TK normalization was performed as described in Shimada et al. [80].

## Supporting Information

Figure S1 The functionality of the tagged Arp6 and Swr1 was confirmed by monitoring cell growth and sensitivity to hydeoxyurea (HU). Five-fold serial dilutions of each strain were plated on YPD with or without 50 mM HU and incubated at 30°C or 37°C for 3 days.
(1.09 MB TIF)

Figure S2 Localization of Arp6 and Swr1 on chromosome 3. The binding of Arp6-FLAG (top), Swr1-FLAG (middle), and Arp6-FLAG in *swr1* cells (bottom) are compared. The position of Tel 3L, Tel 3R, *CEN3*, and the RP gene are shown under the panels.
(9.62 MB TIF)

Figure S3 Localization of Arp6 and Swr1 on chromosome 4. The binding of Arp6-FLAG (top), Swr1-FLAG (middle), and Arp6-FLAG in *swr1* cells (bottom) in the whole chromosome region are compared. The position of Tel 4L, Tel 4R, *CEN4*, *SWR1*, and RP genes are shown under the panels.
(2.67 MB TIF)

Figure S4 Localization of Arp6 and Swr1 on the region including the *SWR1* gene of chromosome 4. The binding of Arp6-FLAG (top), Swr1-FLAG (middle), and Arp6-FLAG in *swr1* cells (bottom) are compared. The position and orientation of the *SWR1* gene is shown.
(1.86 MB EPS)

Figure S5 Localization of Arp6 and Swr1 on chromosome 5. The binding of Arp6-FLAG (top), Swr1-FLAG (middle), and Arp6-FLAG in *swr1* cells (bottom) are compared. The position of Tel 5L, Tel 5R, *CEN5*, and the RP genes are shown under the panels.
(5.97 MB TIF)

Figure S6 Preferential localization of Arp6 and Swr1 in the 5′ end of genes. Vertical bars represent the binding ratio of proteins in each locus. The binding of Arp6-Flag (Top), Swr1-Flag (middle), and Arp6-Flag in *swr1* cells (bottom) in the region 228K-244K of Chr 6R were compared. The orientation of transcription of the genes of Watson strand and Crick strand in the region was shown by arrows in the map over the panels. Regions of divergent promoters are indicated with gray shadow.
(1.29 MB EPS)

Figure S7 Correlation of the localizations of Arp6 and Swr1. The Arp6-binding log2 ratios of Arp6-binding loci (change *p*-value <0.025) in wild-type (A) and in *swr1* cells (B) are represented as scatterplots versus the Swr1 binding log2 ratio in each Arp6 binding locus of wild-type cells. The yellow lines represent the hypothetical pattern of the data if Arp6 and Swr1 bind equally on the chromosomes.
(2.49 MB EPS)

Figure S8 ChIP analysis for Htz1 in cells lacking Arp6 or Swr1. Htz1 association to the promoter of *GAL1*, *SWR1*, and ribosomal protein (*RPL13A* and *RPS16B*) genes was analyzed using ChIP with an anti-Htz1 antibody (abcam, ab4626) and quantified using real-time quantitative PCR in wild-type (WT), *arp6*, and *swr1* cells. The values are indicated as percentage of input DNA obtained by ChIP with anti-Htz1 antibody. The data points represent the mean ± SD for at least three independent experiments.
(0.90 MB EPS)

Figure S9 Quantitative analysis of *RDS1* (YCR106W) and *UBX3* (YDL091C) in *arp6*- and *htz1*-deletion mutants. The same amount of total RNA from wild-type, *arp6*, and *htz1* cells was analyzed using real-time quantitative RT–PCR. The *ACT1* gene was analyzed as a control. The relative amount of the transcript of the genes to *ACT1* is shown. The data points represent the mean ± SD for at least three independent experiments.
(0.87 MB EPS)

Figure S10 ChIP analysis for nuclear pore complex with *GAL1* gene in *arp6* cells. The association of *GAL1* gene with NPC was analyzed using ChIP with an antibody against nuclear pore complex proteins (Mab414, abcam, ab24609) in wild-type (WT) and *arp6* cells grown on the glucose- or galactose containing media. Immunoprecipitated DNA was quantified using real-time PCR probed for *GAL1* gene. The percentage of recovered DNA over input is plotted relative to wild-type cells on glucose as 1. The data points represent the mean ± SD for at least three independent experiments.
(0.85 MB EPS)

Table S1 Presence of Arp6 in nonrepetitive 10 kb subtelomere zones.
(0.05 MB DOC)

Table S2 Microarray analysis in *arp6Δ* and *swr1Δ* cells.
(1.10 MB XLS)

Table S3 Binding of Arp6 and Swr1 on ribosomal protein genes.
(0.04 MB DOC)

Table S4 Expression of RP genes in *arp6Δ* and *swr1Δ* cells.
(0.04 MB XLS)

Table S5 Genes markedly down-regulated in arp6 cells.
(0.09 MB DOC)

Table S6 Strains used in this study.
(0.06 MB DOC)

Text S1 Primer sequences.
(0.04 MB DOC)
