# Supplementary figures and images for: Actin-Related Protein Arp6 Influences H2A.Z-Dependent and -Independent Gene Expression and Links Ribosomal Protein Genes to Nuclear Pores
Source: PLoS Genet. 2010 Apr 15;6(4):e1000910. doi: 10.1371/journal.pgen.1000910 (PMC2855322; doi:10.1371/journal.pgen.1000910)

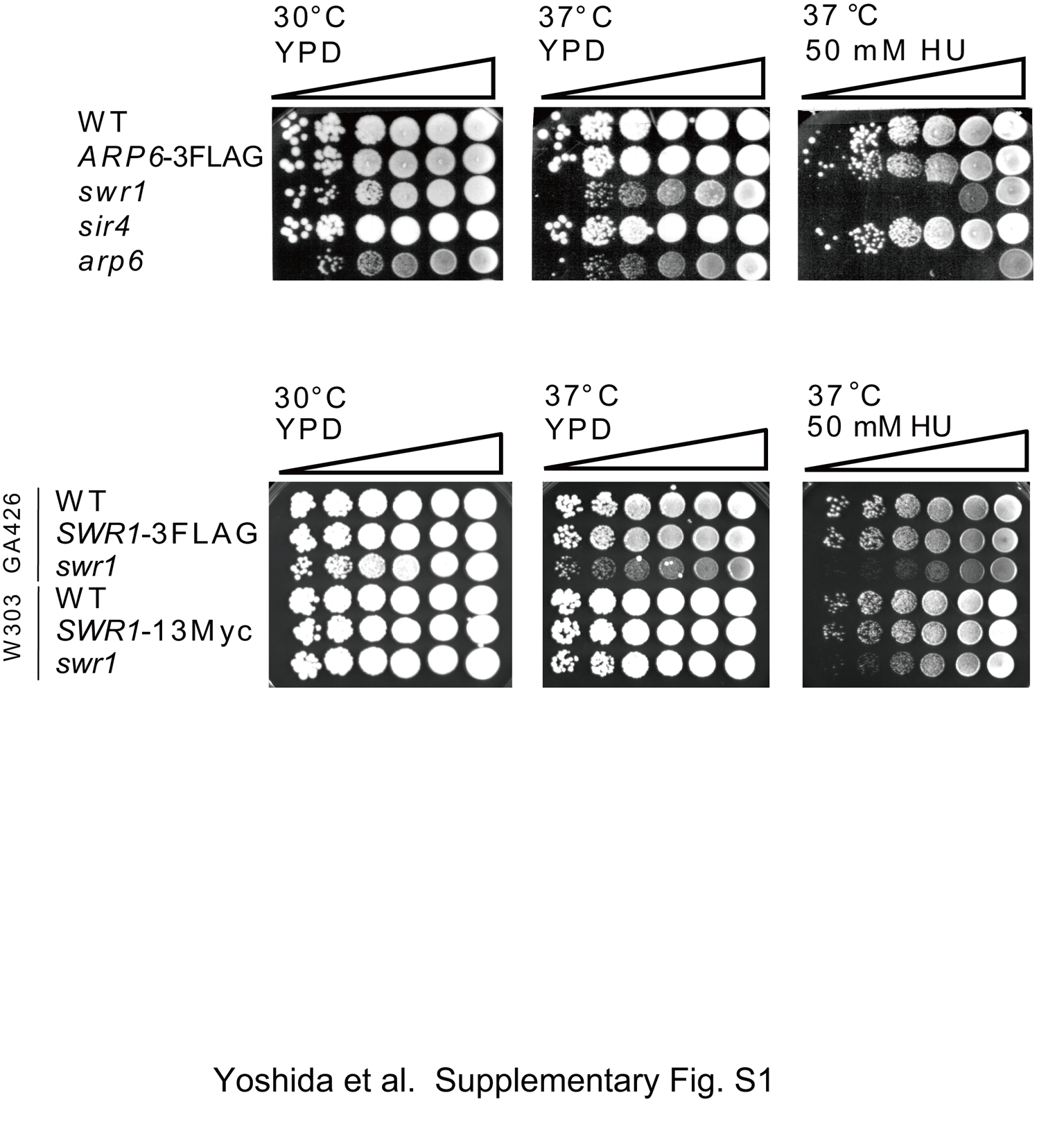

Supplement: Figure S1 — The functionality of the tagged Arp6 and Swr1 was confirmed by monitoring cell growth and sensitivity to hydeoxyurea (HU). Five-fold serial dilutions of each strain were plated on YPD with or without 50 mM HU and incubated at 30°C or 37°C for 3 days. (1.09 MB TIF) [file pgen.1000910.s001.tif]

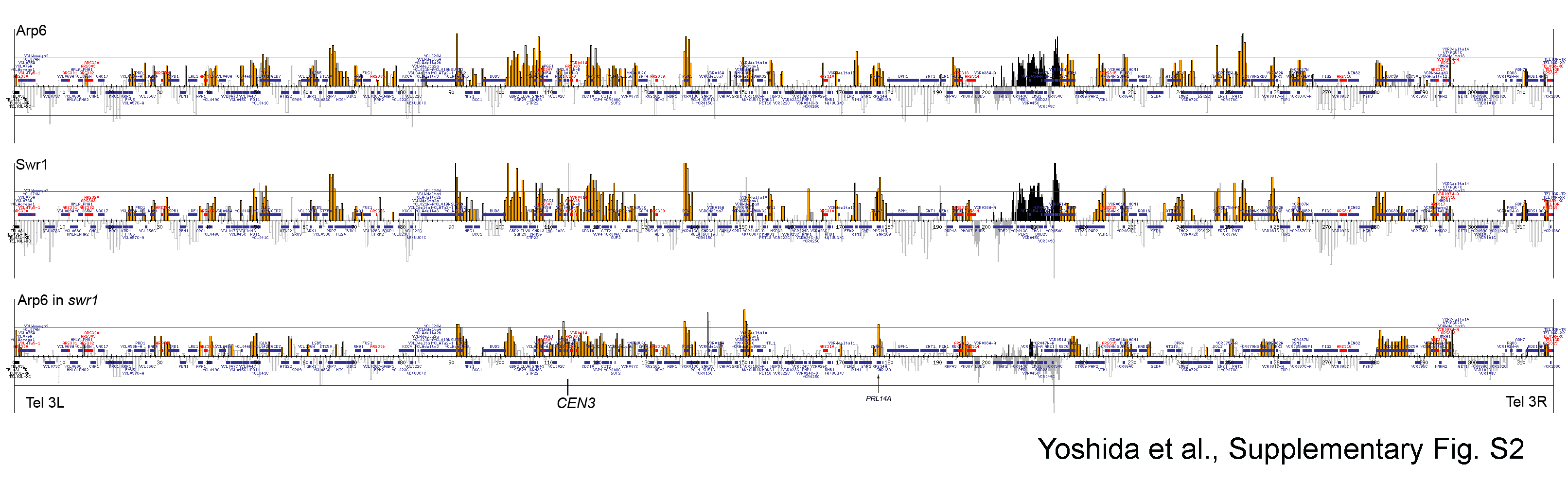

Supplement: Figure S2 — Localization of Arp6 and Swr1 on chromosome 3. The binding of Arp6-FLAG (top), Swr1-FLAG (middle), and Arp6-FLAG in swr1 cells (bottom) are compared. The position of Tel 3L, Tel 3R, CEN3, and the RP gene are shown under the panels. (9.62 MB TIF) [file pgen.1000910.s002.tif]
