# Supplementary material for: Actin-Related Protein Arp6 Influences H2A.Z-Dependent and -Independent Gene Expression and Links Ribosomal Protein Genes to Nuclear Pores
Source: PLoS Genet. 2010 Apr 15;6(4):e1000910. doi: 10.1371/journal.pgen.1000910 (PMC2855322; doi:10.1371/journal.pgen.1000910)
Supplement: Table S1 — Presence of Arp6 in nonrepetitive 10 kb subtelomere zones. (0.05 MB DOC) [file pgen.1000910.s011.doc]

**Supplementary Table S1.** Presence of Arp6 in nonrepetitive 10 kb subtelomere zones

|  |  | Chr3L (32)^1)^  Chr3R (34) | Chr4L (4)  Chr4R (13) | Chr5L (15)  Chr5R (13) | Chr6R (34) | Total (145) |
| --- | --- | --- | --- | --- | --- | --- |
| *SWR1* | Arp6 binding loci | 1^2)^ (3%)^3)^  3 (9%) | 1 (25%)  1 (8%) | 3 (20%)  0 (0%) | 7 (21%) | 16 (11%) |
|  | coincidence with Swr1 binding | 0 (0%)  3 (9%) | 0 (0%)  0 (0%) | 2 (13%)  0 (0%) | 2 (6%) | 7 (5%) |
| *swr1* | Arp6 binding loci | 3 (9%)  4 (12%) | 0 (0%)  5 (38%) | 0 (0%)  1 (8%) | 9 (26%) | 22 (15%) |

1) number of subtelomeric probes in the array, 2) number of probes on which the ChIP-chip signal is significantly positive, 3) percentage of binding-positive probes
