# Supplementary material for: Actin-Related Protein Arp6 Influences H2A.Z-Dependent and -Independent Gene Expression and Links Ribosomal Protein Genes to Nuclear Pores
Source: PLoS Genet. 2010 Apr 15;6(4):e1000910. doi: 10.1371/journal.pgen.1000910 (PMC2855322; doi:10.1371/journal.pgen.1000910)
Supplement: Table S3 — Binding of Arp6 and Swr1 on ribosomal protein genes. (0.04 MB DOC) [file pgen.1000910.s013.doc]

**Supplementary Table S3. Binding of Arp6 and Swr1 on ribosomal protein genes**

|  | | Chr3 (1) | Chr4 (18) | Chr5 (6) | Chr6R (2) | Total (27) |
| --- | --- | --- | --- | --- | --- | --- |
| *SWR1* | Arp6 binding | 1 (100%) | 17 (94%) | 6 (100%) | 1 (50%) | 25 (93%) |
|  | Swr1 binding | 1 (100%) | 16 (89%) | 6 (100%) | 1 (50%) | 24 (89%) |
| *swr1* | Arp6 binding | 1 (100%) | 17 (94%) | 6 (100%) | 2 (100%) | 26 (96%) |
