# Supplementary material for: Actin-Related Protein Arp6 Influences H2A.Z-Dependent and -Independent Gene Expression and Links Ribosomal Protein Genes to Nuclear Pores
Source: PLoS Genet. 2010 Apr 15;6(4):e1000910. doi: 10.1371/journal.pgen.1000910 (PMC2855322; doi:10.1371/journal.pgen.1000910)
Supplement: Table S5 — Genes markedly down-regulated in arp6 cells. (0.09 MB DOC) [file pgen.1000910.s015.doc]

**Supplementary Table S5. Genes markedly down-regulated in *arp6*** cells

|  |  | |  | | |  |  | |  |
| --- | --- | --- | --- | --- | --- | --- | --- | --- | --- |
| ORF name | | *arp6*/wt  log2 ratio | |  | Gene name | | | Description | |
| YNR034W-A | | -4.32 | |  |  | | | Hypothetical protein | |
| YOL052C-A | | -4.16 | |  | DDR2 | | | Induced by DNA damage, heat shock, osmotic shock, etc | |
| YDR070C | | -3.90 | |  | FMP16 | | | Hypothetical protein | |
| YBR072W | | -3.85 | |  | HSP26 | | | Heat shock protein 26 | |
| YHR139C | | -3.73 | |  | SPS100 | | | Sporulation-specific wall maturation protein | |
| YGR088W | | -3.31 | |  | CTT1 | | | Cytoplasmic catalase T | |
| YMR105C | | -3.21 | |  | PGM2 | | | Phosphoglucomutase | |
| YGR052W | | -3.16 | |  | FMP48 | | | similarity to ser/thr protein kinases | |
| YCR021C | | -3.11 | |  | HSP30 | | | Induced by heat shock, ethanol treatment | |
| YDR281C | | -3.09 | |  | PHM6 | | | Unknown function, expression is regulated by phosphate levels | |
| YGR248W | | -3.05 | |  | SOL4 | | | Similar to SOL3 | |
| YML128C | | -2.95 | |  | MSC1 | | | Unknown function | |
| YMR175W | | -2.95 | |  | SIP18 | | | Induced by osmotic stress | |
| YFL014W | | -2.92 | |  | HSP12 | | | 12 kDa heat shock protein | |
| YMR250W | | -2.82 | |  | GAD1 | | | Glutamate decarboxylase, involved in oxidative stress | |
| YBR050C | | -2.74 | |  | REG2 | | | Putative Glc7 regulatory subunit | |
| YMR107W | | -2.67 | |  | SPG4 | | | Required for survival at high temperature | |
| YBR285W | | -2.66 | |  |  | | | Hypothetical protein | |
| YMR206W | | -2.63 | |  |  | | | Weak similarity to hypothetical protein YNR014w | |
| YHR140W | | -2.61 | |  |  | | | Hypothetical protein | |
| YGR256W | | -2.60 | |  | GND2 | | | Zink-finger 6-phosphogluconate dehydrogenase | |
| YPL054W | | -2.57 | |  | LEE1 | | | Unknown function | |
| YEL011W | | -2.50 | |  | GLC3 | | | 1,4-glucan-6-(1,4-glucano)-transferase | |
| YLR178C | | -2.47 | |  | TFS1 | | | Lipid binding protein (putative) | |
| YER103W | | -2.46 | |  | SSA4 | | | Member of 70 kDa heat shock protein family | |
| YPL223C | | -2.42 | |  | GRE1 | | | Hydrophilin of unknown function, stress-induced | |
| YPR192W | | -2.40 | |  | AQY1 | | | Aquaporin | |
| YLL026W | | -2.39 | |  | HSP104 | | | 104 kDa heat shock protein | |
| YOR186W | | -2.38 | |  |  | | | Hypothetical protein | |
| YMR081C | | -2.38 | |  | ISF1 | | | Involved in mRNA splicing | |
| YDL085W | | -2.37 | |  | NED2 | | | Mitochondrial external NADH dehydrogenase | |
| YMR169C | | -2.36 | |  | ALD3 | | | Aldehyde dehydrogenase | |
| YGL156W | | -2.31 | |  | AMS1 | | | Vacuolar alpha mannosidase | |
| YLL052C | | -2.25 | |  | AQY2 | | | Aqy2p, putative aquaporin, member of MIP family | |
| YGL256W | | -2.24 | |  | ADH4 | | | Alcohol dehydrogenase isoenzyme IV | |
| YDL022W | | -2.24 | |  | GPD1 | | | Glycerol-3-phosphate dehydrogenase | |
| YJL163C | | -2.23 | |  |  | | | Hypothetical protein | |
| YDR247W | | -2.22 | |  | VHS1 | | | Cytoplasmic serine/threonine protein kinase | |
| YOL083W | | -2.22 | |  |  | | | Similarity to YOL082w | |
| YER067W | | -2.20 | |  |  | | | Strong similarity to hypothetical protein YIL057c | |
