# Supplementary material for: Actin-Related Protein Arp6 Influences H2A.Z-Dependent and -Independent Gene Expression and Links Ribosomal Protein Genes to Nuclear Pores
Source: PLoS Genet. 2010 Apr 15;6(4):e1000910. doi: 10.1371/journal.pgen.1000910 (PMC2855322; doi:10.1371/journal.pgen.1000910)
Supplement: Table S6 — Strains used in this study. (0.06 MB DOC) [file pgen.1000910.s016.doc]

**Supplementary Table S6. Strains used in this study**

| Name | Genotype |
| --- | --- |
| GA-426 | *MAT*a *ade2::*hisG *his3-11 leu2 trp1 ura3-52 can1::*hisG *VR::ADE2-TEL* |
| GA-3353 | GA-426 *arp6::ARP6-3FLAG-Kan* |
| GA-3354 | GA-3353 *swr1::TRP1* |
| GA-3153 | GA-426 *swr1::SWR1-3FLAG-Kan* |
| GA-3477 | GA-426 *sir4::SIR4-x3FLAG-Kan* |
| GA-1320 | *MAT*a *ade2-1 can1-100 his3-11,-15::GFP-LacI-HIS3 trp1-1, ura3-1, leu2-3,-112 nup49::NUP49-GFP-URA3* |
| GA-1461 | GA-1320 *PES4*::lacO-lexA-*TRP1* (integrated 272 bp 5’ of *PES4*). |
| GA-3320 | GA-1461 *swr1::CaURA3* |
| GA-2765  GA-2659  GA-4135 | GA-1461 *mlp1::KanMX6 mlp2::CaURA3*  GA-1320 *LYS2*::lacO-lexA-*TRP1*  *MATalpha hml::ADE1 hmr::ADE1 ade3::GALHO*  *nup133::NUP133-13myc-Kan* |
| GA-5806 | GA-4135 *arp6::TRP1* |
| GA-5807 | GA-4135 *swr1::TRP1* |
| GA-5868 | GA-3353 *htz1*::*TRP1* |
| GA-3150 | *arp6::ARP6-3FLAG-Kan ade2-1 can1-100 his3-11,-15 trp1-1 ura3-1, leu2-3,-112* |
| GA-3203 | GA-3150 *swr1::CaURA3* |
| GA-3426  GA-3427  GA-3428  GA-3429  GA-3430  GA-4584  GA-3635  GA-5113  GA-5132  GA-4098  GA-6024 | GA-3425 *arp6::KanMX6*  GA-3425 *swr1::CaURA3*  *MAT*a, *ade2-1 can1-100 his3-11,-15 trp1-1 ura3-1 leu2-3,-122 ino80::INO80-13MYC-HIS3MX6*  GA-3428 *arp6::KanMX6*  GA-3428 *swr1::CaURA3*  *MAT*a *ade2-1 ::GFP-LacI-ADE2 can1-100 his3-11,-15 trp1-1, ura3-1, leu2-3,-112 nup49::NUP49-CFP-URA3 LYS2::LacO-LexA-TRP1 nup133::HIS3 pUN100-nup133ΔN-KanMX6*  GA-1320 with *RPL9A::LacO-TRP1*  GA-3635 with *swr1::CaURA3*  GA-3635 with a*rp6::CaURA3*  GA-1320 with *GAL10::LacO-TRP1*  GA-4098 with *arp6::CaURA3* |
