# Supplementary material for: Actin-Related Protein Arp6 Influences H2A.Z-Dependent and -Independent Gene Expression and Links Ribosomal Protein Genes to Nuclear Pores
Source: PLoS Genet. 2010 Apr 15;6(4):e1000910. doi: 10.1371/journal.pgen.1000910 (PMC2855322; doi:10.1371/journal.pgen.1000910)
Supplement: Text S1 — Primer sequences. (0.04 MB DOC) [file pgen.1000910.s017.doc]

**Text S1. Supplementary Materials
Primer sequences**

For *RPS16B*:

forward primer, TCACTGGTGGTGGTCATGTT; reverse primer, CGTTCTTGGATTGTTCGTCA

For *RPL13A*:

forward primer, GCACTGGCAAGAACGTGTTA; reverse primer, GCAGCAGCAGACAAAACTTG

For *RPP1A*:

forward primer, TGGCTGACTCTGAAATCGAA; reverse primer, CGTCCAAAGCCTTAGCAAAA

For *RPL31A*:

forward primer, GCTCCAGAATTGAACCAAGC; reverse primer, TGGCGTCTTCTTCTTCGTTT

For *RPL2A*:

forward primer, ACCTCCCACACCAGATTGAG; reverse primer, CTACCGGAGTCGTGGACAAT

For *RPL29*:

forward primer, AACCAAACCAGAAAGGCTCA; reverse primer, GTAGGGCATGCTTGTGGTTT

For *NUP2*:

forward primer, TAAGGTTGCGTCATCTGCTG; reverse primer, CTGCTTGGTTTCATCCGATT

For *FAB1*:

forward primer, GCGACGGATTTGAACACATT; reverse primer, ATATTCGCGCTCTTGCCTAA.

For *PES4*:

forward primer, CGAGCCATTCTGATTCACCT; reverse primer, TGTTTCATGGAGGTCACCAA.

For *GAL1*:

forward primer, GCGCAAAGGAATTACCAAGA; reverse primer, GGCGCAAAGCATATCAAAAT

For *SWR1*:

forward primer, GCTTCCTCTGGTTCAGATGC; reverse primer, AGACAAGGGTTCCGATGATG

For *UBX3*:

forward primer, GGGATGGACGCTTAAAATCA; reverse primer, AAACGGTTGTGCTTCCTCTG

For *RDS1*:

forward primer, GTGATAAACTGCGGCCTGCT; reverse primer, AACGACACCTTCAGGCACTT

For *RPL13A*:

forward primer, TCTGTTCGAAGGGGTTTGAG; reverse primer, TACGATTCCTGCTGTGCTGT

For *RPS16B*:

forward primer, TCACTGGTGGTGGTCATGTT, reverse primer, CGTTCTTGGATTGTTCGTCA
